# The Skin Microbiome of Patients With Atopic Dermatitis Normalizes Gradually During Treatment

**DOI:** 10.3389/fcimb.2021.720674

**Published:** 2021-09-24

**Authors:** Veda D. Khadka, Felix M. Key, Carolina Romo-González, Adrián Martínez-Gayosso, Blanca L. Campos-Cabrera, Armando Gerónimo-Gallegos, Tucker C. Lynn, Carola Durán-McKinster, Rafael Coria-Jiménez, Tami D. Lieberman, Maria T. García-Romero

**Affiliations:** ^1^ Institute for Medical Engineering and Sciences, Massachusetts Institute of Technology, Cambridge, MA, United States; ^2^ Department of Civil and Environmental Engineering, Massachusetts Institute of Technology, Cambridge, MA, United States; ^3^ Experimental Bacteriology Laboratory, National Institute of Pediatrics, Mexico City, Mexico; ^4^ Department of Dermatology, National Institute of Pediatrics, Mexico City, Mexico

**Keywords:** atopic dermatitis (AD), microbiome & dysbiosis, skin, microbiota (16S), therapeutics

## Abstract

**Background:**

Atopic dermatitis (AD) is characterized by an altered skin microbiome dominantly colonized by *S. aureus*. Standard treatment includes emollients, anti-inflammatory medications and antiseptics.

**Objectives:**

To characterize changes in the skin microbiome during treatment for AD.

**Methods:**

The skin microbiomes of children with moderate-to-severe AD and healthy children were investigated in a longitudinal prospective study. Patients with AD were randomized to receive either standard treatment with emollients and topical corticosteroids or standard treatment with the addition of dilute bleach baths (DBB) and sampled at four visits over a three-month period. At each visit, severity of AD was measured, swabs were taken from four body sites and the composition of the microbiome at those sites was assessed using 16S rRNA amplification.

**Results:**

We included 14 healthy controls and 28 patients. We found high relative abundances of *S. aureus* in patients, which correlated with AD severity and reduced apparent alpha diversity. As disease severity improved with treatment, the abundance of *S. aureus* decreased, gradually becoming more similar to the microbiomes of healthy controls. After treatment, patients who received DBB had a significantly lower abundance of *S. aureus* than those who received only standard treatment.

**Conclusions:**

There are clear differences in the skin microbiome of healthy controls and AD patients that diminish with treatment. After three months, the addition of DBB to standard treatment had significantly decreased the *S. aureus* burden, supporting its use as a therapeutic option. Further study in double-blinded trials is needed.

## Introduction

Atopic dermatitis (AD) is the most common chronic inflammatory skin disease characterized by eczematous skin lesions and pruritus in specific body sites, affecting 10-30% children (Bieber, 2008; Bylund et al., 2020). Its complex and multifactorial etiology is driven by a combination of genetic, environmental, and immune factors that include epidermal abnormalities which lead to a defective stratum corneum; enhanced allergen penetration and immunoglobulin E (IgE) sensitization, hyperreactive immune responses; and an altered skin microbiota (Bieber, 2008; Leung and Guttman-Yassky, 2014; Eyerich et al., 2015; Brussow, 2016; Paller et al., 2019).

The relationship between an altered skin bacterial microbiome and AD is well recognized clinically. In recent years, new methods and techniques to study bacteria on and in skin have permitted investigation at the level of species and strains, allowing for more granular insight into the relationship between altered skin flora and disease.

Studies using traditional culturing, 16s rRNA gene sequencing, and shotgun metagenomics have demonstrated a significant increase in the absolute and relative abundance of the opportunistic pathogen *Staphylococcus aureus* during AD flares (Kong et al., 2012; Gonzalez et al., 2016; Totte et al., 2016; Bjerre et al., 2017). Studies using the latter two approaches have also shown a reduction in alpha diversity as assessed by standard microbiome alpha diversity metrics (Kong et al., 2012; Gonzalez et al., 2016; Bjerre et al., 2017). While diversity metrics are sensitive to blooms of bacteria (e.g. *S. aureus*) (Gloor et al., 2017), there are reasons to think that a more diverse microbiota may play a role in mitigating AD severity. In particular, *in vitro* and *in vivo* experiments have shown that various species present on healthy skin, including the ubiquitous *Staphylococcus epidermidis*, can generate anti- *S. aureus* responses through microbe-microbe and microbe-host interactions, including modulation of host immune responses (Iwase et al., 2010; Lai et al., 2010; Park et al., 2011).

Targeting the skin bacterial community using antibiotic cocktails reduces the relative abundance of *S. aureus*, increases diversity, and improves eczematous lesions dramatically in mouse models (Kobayashi et al., 2015). In clinical studies, decreasing burden or colonization of *S. aureus* using antimicrobial treatments has also been demonstrated to improve AD severity (Huang et al., 2009; Kobayashi et al., 2015). However, sustained antibiotic use for AD management and prevention is not practical long-term, as it can result in undesirable effects on commensal skin bacteria, on the microbiota in the gut and other sites, and it can contribute to antibiotic resistance, an increasing public health problem (Harkins et al., 2018). For this reason, other anti-*S.aureus* therapeutic options are worth exploring, such as dilute bleach baths (DBB).

Standard treatment for AD includes the use of emollients to improve the skin barrier, varying combinations of topical or systemic corticosteroids, anti-inflammatory or immunosuppressive medications, antibiotics, and dilute sodium hypochlorite (house bleach) baths. The antibacterial properties of bleach are thought to be mediated by the generation of superoxide radicals by hypochlorous acid, which can cause oxidative injury and bacterial cell death (Barnes and Greive, 2013). DBB may offer a benefit to patients by other mechanisms of action as well (Ryan et al., 2013; Wong et al., 2013; Leung and Guttman-Yassky, 2014; Brussow, 2016). Several clinical studies have reported varying effects on disease severity, microbiome composition, and *S. aureus* abundance when standard AD treatment was supplemented with DBB, although these studies varied widely in their methodologies and assessment metrics (Metry et al., 2007; Fritz et al., 2011; Wong et al., 2013; Gonzalez et al., 2016; Chopra et al., 2017).

Here, we used high-throughput sequencing to study the dynamics of the skin bacterial microbiome during the course of disease in pediatric AD patients with moderate-to-severe disease, to compare with healthy controls, and to better understand the effect of treatment with or without DBB.

## Materials and Methods

### Patient Enrollment

This randomized non-blinded study was approved by the Institutional Review Board of the National Institute for Pediatrics, Mexico City (registration no. 042/2016) and Massachusetts Institute of Technology (MIT), Cambridge, and was conducted according to the Declaration of Helsinki principles. Patients with moderate to severe AD and age-matched healthy children who attended the Dermatology Clinic at the NIP were recruited. Children aged 5 to 18 years with the diagnosis of AD were included in this study if they had AD as defined by the modified Hanifin and Rajka criteria (Hanifin and Rajka, 1980), had moderate or severe disease according to the SCOring Atopic Dermatitis (SCORAD) clinical tool score ≥ 25, had not received topical or systemic antibiotics for the past month, and provided written consent to participate. Healthy controls were children aged 5 to 18 years without any systemic inflammatory or autoimmune diseases, who had not received topical or systemic antibiotics for the past month (Kong et al., 2012) and were not being currently treated with any systemic medication and provided written consent to participate. Exclusion criteria included later diagnosis of other inflammatory or autoimmune conditions, treatment with systemic immunomodulatory drugs or corticosteroids, and/or actual visit dates not within 30 days of the monthly planned visit schedule. Our sample size of a minimum of 12 subjects per group was determined due to convenience, comparability with the literature, and because this study was part of a more intensive effort of tracking S. aureus evolution on individual patients (Key et al., 2021).

**Figure 1:**
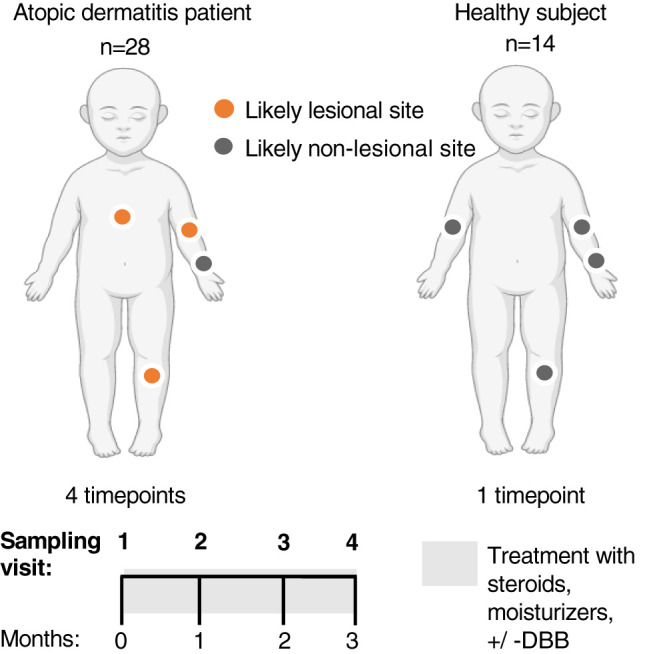
Longitudinal unblinded study of standard treatment *vs* standard treatment + dilute bleach baths (DBB) in children with Atopic Dermatitis (AD). 28 children with AD were sampled at four sites (right anterior forearm, right antecubital and popliteal fossa and another lesional site chosen to represent the area of worst active AD on that patient) across four visits, spanning three months. The forearm was determined to be a likely non-lesional site on AD patients. 14 healthy controls were also recruited and sampled similarly (both antecubital fossae, popliteal fossa and forearm) at a single visit. This figure was created with BioRender.com.

After providing written consent from parent(s) and assent by children older than 12 years of age, patients and healthy controls were enrolled between June 2017 and December 2018. Patients were sampled at four timepoints (baseline during flare, and one, two, and three months later). Some deviation from this schedule occurred due to patients’ schedules, as well the disruption caused by the earthquake in September 2017. Healthy controls were sampled once (**Figure 1**).

### Study Procedure

Disease severity in patients was measured using SCORAD. Patients were prescribed standard treatment with class II to VI topical corticosteroids (TCS) twice daily until improvement according to the location and severity of their AD, with or without DBB according to block randomization (ratio 1:1) using sequentially numbered envelopes previously generated by an independent individual and concealed to investigators who enrolled patients (MG-R and BC-C) and assigned to interventions (AM-G). Those randomized to the DBB condition were instructed to add 1 mL of 6% liquid house bleach per litre of bath water to achieve a concentration of 0.006%, and to soak twice weekly for 10-15 minutes. All patients were instructed to use mild soap for cleansing daily and to use bland emollients twice or thrice a day as part of the recommended skin routine for AD. Patients were instructed to avoid bathing for 24 hours prior to subsequent visits. Subjects were instructed not to take antibiotics during the study and were not instructed regarding probiotics.

Superficial samples were taken from four sites to represent the range of commonly affected and unaffected AD sites: right anterior forearm (as an unaffected non-lesional site), right antecubital and popliteal fossa (as commonly affected lesional AD sites) and an actively lesional AD site from patients (**Figure 1**). From subjects 1-4, bilateral samples were taken to understand variability, and replicate swabs were taken at each site. For each site, a new sterile applicator was moistened in sterile TES buffer (10mM Tris-HCl; 1mM EDTA; 100mM NaCL) and used to swab the skin at the specified site 40 times over a five cm^2^ area, pressing firmly and twirling the swab to coat all surfaces. The applicator was then placed into a microtube with 0.5 mL of TES buffer and rotated against the side of the vial to release any biomaterial present. Immediately after sampling, samples were labeled and frozen at -20°C until shipment and further processing.

At each subsequent visit SCORAD was measured, and samples were taken as above. If control of disease had been achieved, patients were instructed to continue using TCS twice weekly as proactive treatment in previously affected areas +/- DBB twice weekly as indicated. Granular information about each subject is included in **Supplementary Table 1**.

### DNA Extraction and Sequencing

DNA was extracted in the collection buffer using ReadyLyse (Lucigen) at a final concentration of 1250 U/ul and incubated at room temperature for 12 hours. The V1-V3 region of the bacterial 16s rRNA gene was amplified for 36 cycles using 27F-plex (TCGTCGGCAGCGTCAGATGTGTATAAGAGACAGAGAGTTTGATCMTGGCTCAG) and 534R-plex (GTCTCGTGGGCTCGGAGATGTGTATAAGAGACAGATTACCGCGGCTGCTGG) the KAPA HiFi HotStart ReadyMix with an annealing temperature of 54.5C. Two independent PCR replicates were performed and barcoded separately for each lysis product. Samples were cleaned using a bead-based approach (Rohland and Reich, 2012) and a round of barcoding PCR was performed for 14 cycles using standard primers (Baym et al., 2015). Samples were cleaned again and sequenced on an Illumina MiSeq with 300 paired-end sequencing to a median number of 16,007 reads (considering each replicate separately).

### 16S rRNA Data Processing and ASV Classification Using QIIME2

All data processing was done using QIIME2 (v2019.01) (Bolyen et al., 2019), including de-multiplexing and primer removal. Forward reads were filtered for a minimum base quality (default: –p-min-quality 4) and truncated; and removed if the retained sequence was below < 75% (default) of the input sequence. Reverse reads were not used because the quality was too low to overlap read pairs. All sequences were denoised using the deblur algorithm (Amir et al., 2017)(trimmed to 209bp) to generate the feature [amplicon sequence variant (ASV)] table. ASVs with less than 10 representative reads were removed leading to 7,913 ASVs in total.

We built a custom classifier for ASVs based on a cleaned-up version of the SILVA database (version 132) (Quast et al., 2013). We extracted target sequences from SILVA based on the primers used here and retained all sequences 100-700 bp. Erroneous taxonomic sequence labels in a 16S rRNA dataset can prevent species identification despite sufficient nucleotide information present in the amplified fragment of the 16S rRNA gene. Here, to ensure correct identification of species within the *Staphylococcus* genus, we removed: (i) 1 sequence with a non-species taxon assignment, (ii) 25 taxa that had ≤ 10 assigned sequences, or (iii) 12 taxa where > 60% of sequences were identical to another taxa. Critically, sequences with taxonomic misclassifications in the database were removed using the phylogeny-aware pipeline SATIVA (Kozlov et al., 2016). SATIVA removed 69 sequences out of the 17,882 (0.39%) *Staphylococcus* sequences in the SILVA database. The filtered data was used to train the naive bayes classifier in QIIME2. The resulting ASVs were exported and taxa-level assignments used for further analysis.

Exported taxa from QIIME2 analyses were analyzed in R version 3.3.3. We identified potential contaminant sequences in these low biomass samples by looking for sequences that were specific to either sequencing replicate, as each replicate was run in an independent batch. We performed principal component analysis (PCA) to identify taxa that were segregated by batch: a high relative abundance of Delftia in one batch and Pseudomonas in another. These two genera are usually not associated with skin microbiome composition (Byrd et al., 2017), and are known common contaminants (Salter et al., 2014). We removed both taxa and an additional seven taxa that showed high covariance (> 0.34) with Delftia across all samples (*Serratia*, *Stenotrophomonas*, *Microbacterium*, *Ralstonia*, *Pelomonas*, *Microbacterium testaceum*, *Methylobacterium*). We also removed any Cyanobacteria. Samples were removed from downstream analysis if they had fewer than 500 reads after this filtering or if greater than 25% of reads matched the pool-based contaminants. We then merged samples across replicate pools if pools had a Bray-Curtis distance of less than 0.75; more divergent pools were deemed to be of low quality and discarded (removed 17.13% of samples at this stage). Replicate swab samples from the more intensively sampled subjects were merged after this step. Critically, we did not evaluate outcomes when setting these quality thresholds to avoid p-value hacking.

### Statistical Analyses

Subjects were included in the longitudinal analysis if their actual visit dates were within 30 days of the monthly planned visit schedule (timepoints excluded = 6). Data was analyzed using the intention-to-treat principle. Correlation analyses were performed using Spearman’s correlation, comparisons between groups were done using the Wilcoxon Rank-Sum test and principal coordinate analysis (PCoA) was performed on Bray-Curtis dissimilarity using phyloseq (McMurdie and Holmes, 2013), and vegan (Oksanen et al., 2017) packages. Code used for analyses is available at: github.com/vedomics/SkinMicrobiomeAD_Analysis. P-values ≤ 0.05 were considered significant.

All sequencing data is available here: http://www.ncbi.nlm.nih.gov/bioproject/759575.

## Results

We included 28 patients and 14 healthy controls (**Supplementary Figure 1**). Twenty-five patients (89%) completed four visits, though data from seven visits that did not fit the anticipated schedule were removed from longitudinal analyses. In total, 540 samples were analyzed. Demographic and clinical characteristics can be found in **Table 1**. Patients in both treatment groups had similar characteristics and baseline SCORAD values.

**Table 1:** Demographic and clinical characteristics of healthy controls and patients included in the study.

	Healthy controls	Patients with AD			P
		All	Standard treatment	Standard treatment + DBB	
n	14	28*	14*	14*	
Female gender	5 (35.7%)	16 (57.1%)	7 (53.8%)	8 (66.6%)	0.64
Age in years, median (range)	10 (5-16)	11.5 (5-15)	12 (5-15)	11 (6-13)	0.14
Years since AD diagnosis, median (range)		3 (1-14)	3 (0-14)	2 (1-13)	
Baseline SCORAD, median (range)		45.10 (25.8-85.6)	46.85 (26.5-84)	44.1 (25.8-85.6)	0.47
Visit 2 SCORAD, median (range)		25.05 (4.3-76.2)	25.7 (8.2-76.2)	22.8 (4.3-68.7)	0.85
		*n=26*	*n=13*	*n=13*	
Visit 3 SCORAD, median (range)		22.9 (1-70.9)	26.70 (8.6-70.9)	17.1 (1-58.4)	0.12
		*n=24*	*n=11*	*n=13*	
Visit 4 SCORAD, median (range)		15.90 (0-75.7)	17.9 (3.5-75.7)	15.3 (0-66.6)	0.7
		*n=21*	*n=10*	*n=11*	

*Number of patients at the beginning of the study; AD, atopic dermatitis; DBB, dilute bleach baths.

### *S. aureus* Dominates the Microbiome on Lesional Sites and Correlates With AD Severity

**Figure 2:**
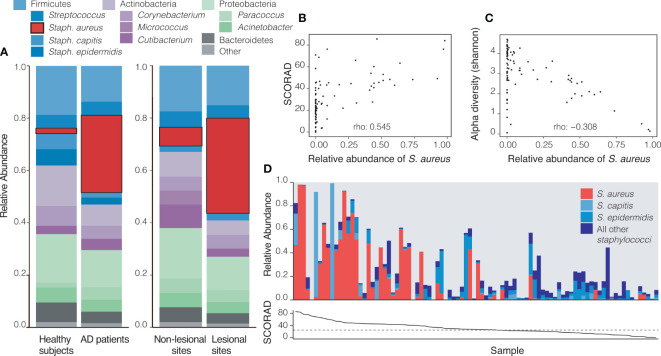
Atopic Dermatitis (AD) sites are dominated by *Staphylococcus aureus*. **(A)** At baseline (visit 1), AD patients have a significantly higher relative abundance of *S. aureus* across all sites (29.56%) when compared to all sites on healthy controls (2.08%, p=1.8 x 10^-6^). Within AD patients, actively lesional sites had a significantly higher relative abundance of *S. aureus* (36.35%) than non-lesional sites (7.20%, p=0.01). **(B, C)** Each datapoint represents an average across all likely lesional and lesional sites from a subject at a given visit (excludes right forearm as a non-lesional site). **(B)** A higher relative abundance of *S. aureus* correlates with higher SCORAD in AD patients (Spearman’s rho = 0.545, p=2.7 x10^-08^). **(C)** *S. aureus* relative abundance inversely correlates with Shannon diversity (rho: -0.307, p=0.003). **(D)** The skin microbiomes of patients with high SCORAD often have a correspondingly higher relative abundance of *S. aureus* and smaller amounts of beneficial Staphylococcus species, though this is not always the case. Top: the relative abundances of *S. aureus*, *S. capitis*, *S. epidermidis* and other staphylococci. Bottom: Corresponding SCORAD values for visits. A dashed line at SCORAD = 25 indicates the cutoff above which disease is considered moderate-severe.

Consistent with expectations from other AD cohorts, a striking pattern in the relative abundance of *S. aureus* at the baseline visit was observed: patients with AD had significantly higher relative abundances of this pathogen both in actively lesional (36.35%) and non-lesional sites (7.20%) compared to all sites on healthy controls (2.08%; p<0.001). Accordingly, healthy controls had non-significantly higher abundances of the health-like flora *Staphylococcus capitis* (p=0.096) and *Micrococcus* sp. (p=0.046) than all sites on AD patients (**Supplementary Table 2** and **Figure 2A**).

The relative abundance of *S. aureus* on likely lesional sites was positively correlated with disease severity as measured by SCORAD (rho = 0.545, p<0.001) (**Figure 2B**). Shannon diversity, an ecological measure of diversity that is heavily influenced by evenness (relative proportion of species) (Strong, 2016), was inversely associated with SCORAD (rho= -0.556, p<0.001, **Supplementary Figure 4B**) and with the relative abundance of *S. aureus* (rho= -0.307, p<0.01) consistent with a model in which an increased abundance of *S. aureus* contributes to lower apparent alpha diversity (**Figure 2C**). In patients with a lower SCORAD, the relative abundance of *S. aureus* across lesional body sites was decreased and that of all other staphylococci showed a trend towards increase (**Figure 2D**). The summed relative abundance of all non- *S. aureus* staphylococcal species was not found to be significantly correlated with SCORAD, although the beneficial species *S. epidermidis* and *S. hominis* were inversely correlated with SCORAD (p<0.05; **Supplementary Figure 3**).

### Treatment Gradually Shifts Bacterial Community Composition in Patients With AD Towards That of Healthy Controls

**Figure 3:**
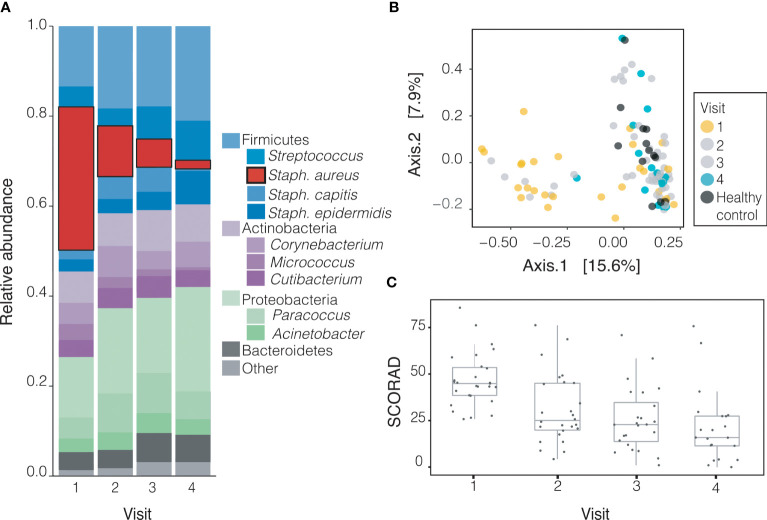
Treatment gradually shifts microbiomes of children with AD towards healthy-like microbiota. **(A)** The relative abundance of *S. aureus* decreases significantly (p=1.4x10^-5^) with treatment from visit 1 (31.83%) to visit 4 (1.90%) across likely lesional sites on AD patients. The decrease in relative abundance of *S. aureus* is significant across treatment groups (+DBB, p<0.001, -DBB, p=0.014, **Supplementary Figure 1**). **(B)** By visit 4 (cyan), the composition of microbiota of likely lesional sites on AD patients is much more similar to healthy controls (dark grey) than at visit 1 (yellow), although there is variation in the severity of disease at both timepoints (PCoA on Bray-Curtis distance). **(C)** Severity of disease as measured by SCORAD decreases with treatment across visits.

In order to better understand longitudinal trends, we analyzed the composition of all sites except the usually unaffected forearm. During the course of the study (from baseline to final visit), the relative abundance of *S. aureus* at these sites decreased significantly over time (31.83% to 1.90%, p<0.001) while that of other species including *S. epidermidis* and members of the genus *Corynebacterium* increased, although none significantly (**Supplementary Table 3** and **Figure 3A**). Principal coordinate analysis (PCoA) on Bray-Curtis distances demonstrates that the bacterial community of children with AD shifted toward that of healthy controls gradually over the course of treatment (**Figure 3B**). Severity of AD as measured by SCORAD decreased over subsequent visits for all subjects (**Figure 3C**).

### Lower Relative Abundance of *S. aureus* in Patients Treated With DBB at the Final Visit

**Figure 4:**
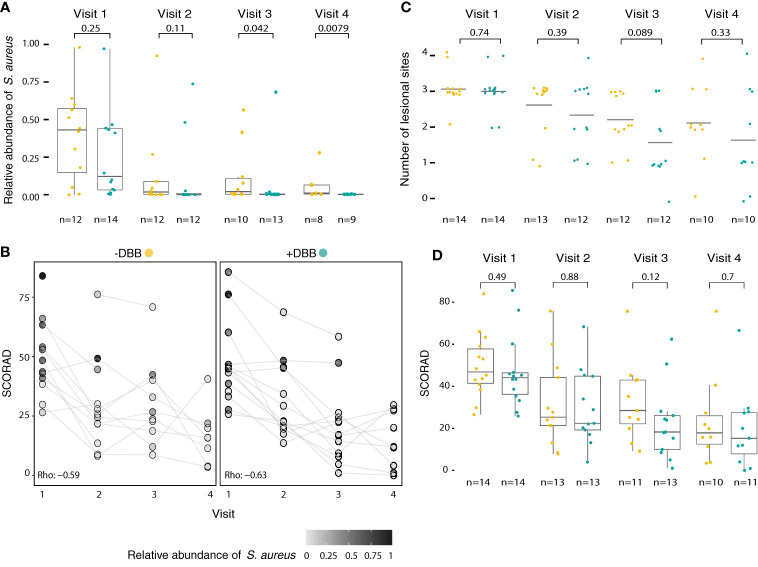
*S. aureus* abundance but not SCORAD is lower in patients after DBB treatment. **(A)** The relative abundance of *S. aureus* decreased a significantly larger amount for patients treated with standard treatment + DBB (cyan) than those receiving standard treatment alone (yellow) after 3 months from initial visit, visit 4 (p=0.01), even though both treatment groups had similar baseline visit relative abundances of S. aureus (+DBB 25.6%; -DBB 39.13%, p=0.25). **(B)** Individual patient trajectories (patients are represented by grey circles, lines indicate SCORAD trajectory and gradient indicates *S. aureus* relative abundance) highlight that patients treated with DBB had larger decreases in the relative abundance of *S. aureus* than the standard treatment group, despite having similar values for both SCORAD and *S. aureus* abundance at the initial visit. In both groups, SCORAD exhibited a decrease over time, as evaluated by a correlation of SCORAD and timepoint (+DBB Spearman’s rho = -0.63, p=1.6 x 10^-6^; –DBB Spearman’s rho = -0.59, p=4x10^-5^). **(C)** The number of actively lesional sites per patient decreases across both treatment groups over time. Both treatment groups display a similar number of actively lesional sites at baseline visit (p=0.74), and have similarly decreased numbers of lesional sites by visit 4, reflecting improvement in condition as indicated by SCORAD. **(D)** In both groups, treatment results in improved patient condition, as indicated by SCORAD. At baseline, both treatment groups present with similar SCORAD values (mean +DBB: 46.6; mean -DBB: 49.6; p=0.49), which decrease with treatment by visit 4 (mean +DBB: 19.5; mean –DBB: 23.6, p=0.38).

We found that patients treated with DBB in addition to standard treatment had significantly less *S. aureus* averaged across all likely lesional sites at visit 4 than the standard treatment group (0.05% *vs* 3.99%; p=0.01) (**Figure 4A**), although both groups showed a decrease in *S. aureus* abundance over time (**Supplementary Figure 2**). In **Figure 4B**, we illustrate individual patient trajectories over time by treatment groups. We note that, at baseline, patients treated with DBB had lower, but not significantly lower, relative abundances of *S. aureus* at these sites (p=0.25). The number of sites affected by AD (**Figure 4C**) and disease severity (**Figure 4D**) decreased over time similarly in both groups, and there was a trend towards lower SCORAD across visits in patients who received standard treatment plus DBB.

## Discussion

To our knowledge, we have performed the most detailed longitudinal study of changes in the skin microbiome during AD treatment. We find that the skin microbiomes of children treated with DBB in addition to standard treatment have a significantly lower abundance of *S. aureus* after 3 months of treatment compared to the standard treatment group (**Figure 4**). Patients treated with DBB also had lower disease severity as measured by SCORAD, though this was not statistically significant. Adjuvant DBBs have been traditionally used to treat patients with AD and reduce disease severity. However, there are conflicting hypotheses regarding the effects and mechanisms of action of DBB on AD. The concentration most often used in AD treatment is 0.005% (0.002-0.016%), and *in vitro* studies have shown that DBB in concentrations as low as 0.005% are effective in reducing *S. aureus* abundance (McKenna et al., 1991; Rutala et al., 1998). Other studies have suggested that bleach changes the expression of virulence factors in *S. aureus* (Sawada et al., 2019) or that it provides a direct anti-inflammatory action (Huang et al., 2009; Leung et al., 2013; Wong et al., 2013). Regardless of their mechanism of action, our findings support the idea that DBB may be beneficial in patients with AD, thus avoiding the harmful effects antibiotic therapy has of altering other body sites´ microbiomes, perturbing the rest of the skin bacterial community (**Supplementary Figure 2D**), or increasing the risk of bacterial resistance. While other studies have also suggested a benefit of DBB in lowering *S. aureus*, our longitudinal study covered a longer, three-month period, allowing us to capture differences in *S. aureus* abundance and SCORAD across treatment groups at later visits.

Our baseline samples confirm previously noted differences between healthy individuals and patients with AD. In particular, we confirm that the skin of patients with AD is characterized by a higher relative abundance of *S. aureus* than healthy controls (Kong et al., 2012; Gonzalez et al., 2016; Byrd et al., 2017). We also confirm that *S. aureus* is more abundant on lesional skin than non-lesional skin, and that a higher abundance of *S. aureus* is correlated with disease severity. In line with these results, previous work has shown that AD patients who are colonized with *S. aureus* have increased Type 2 immune responses and increased disease severity (Simpson et al., 2018). While this and other observational studies cannot disentangle causation from correlation, and a disrupted or inflamed skin barrier may facilitate colonization with pathogenic bacteria, *S. aureus* colonization has been shown to precede the detection of AD development and flares (Kong et al., 2012; Meylan et al., 2017). Moreover, *S. aureus* is known to potentiate skin barrier defects and inflammation through toxins with superantigen properties, toll-like receptor ligands, proteases, surface proteins (Nakamura et al., 2013; Kobayashi et al., 2015; Cau et al., 2020), and by stimulating the proliferation of T cells (Iwamoto et al., 2017).

This study shows with unprecedented longitudinal resolution the recovery process of the skin microbiome during treatment for AD and bolsters findings from other studies (Kong et al., 2012; Gonzalez et al., 2016). As expected, the relative abundance of *S. aureus* decreased gradually in both treatment groups. By the final visit, the relative abundance of *S. aureus* in AD lesional sites had decreased significantly compared to baseline, such that the bacterial microbiome of children with AD became more similar to that of healthy controls after two months of treatment.

We also confirmed previous findings of lower alpha diversity in AD patients relative to controls and an increase of alpha diversity following treatment (Kong et al., 2012; Gonzalez et al., 2016). However, we recommend caution in interpreting this metric, as much of the apparent reduction in alpha diversity in AD patients can be explained by blooms of S. aureus or other taxa (**Supplementary Figure 4**). By its nature, 16S amplicon sequencing measures the fraction of sequencing reads that originate from a given species and not their absolute abundance in the sample. Therefore, all conventional alpha diversity metrics, including Shannon diversity, are confounded when a single species rises in absolute abundance (Byrd et al., 2017; Gloor et al., 2017). We illustrated this problem by removing *S. aureus* reads from our data and recalculating diversity metrics; this approach shows a diminished relationship between AD severity and diversity (**Supplementary Figure 4**). We note that even this analysis does not sufficiently assess community differences between samples, as the low number of sequencing reads that remain after removal of *S. aureus* creates imprecision in measurements of remaining taxa.

Our study had several limitations and potential biases. A relatively small cohort size limited our power to detect differences in clinical outcomes between treatment groups. Emollient and corticosteroid use were not standardized, adding more variation in outcomes between subjects, and we did not characterize host genetics or host disease beyond the use of SCORAD. While subjects were randomized to treatment groups, investigators and patients were not blinded, as subjects self-administered treatment it is possible that those in the DBB group paid more attention to skin care overall. In addition, not every sample was included in our final analysis, due to removal of samples with low sequencing reads after quality control steps (Methods).

In conclusion, this longitudinal study confirmed the association between *S. aureus* and AD severity. We find higher *S. aureus* abundances in patients with AD relative to healthy controls, in lesional sites relative to non-lesional sites, and in patients with higher *vs* lower SCORAD. Moreover, the abundance of *S. aureus* decreases and the skin microbiomes of patients measurably shifts towards that of healthy controls as patients´ disease severity improves with standard treatment. Crucially, we find that the addition of DBB to a standard treatment regimen resulted in significantly lower relative abundances of *S. aureus* and a non-significantly lowered disease severity, generally improving upon standard treatment.

These findings add support to the use of DBB to complement traditional AD therapy. Additional studies, particularly with larger sample sizes, are needed to fully establish the benefit of DBB in treating AD and modifying the skin microbiome.

## Data Availability Statement

The data presented in the study are deposited in the SRA repository, accession number PRJNA759575.

## Ethics Statement

This study involving human participants was reviewed and approved by the National Institute of Pediatrics Research and Ethics Research Board (no. 42/2016) and the Massachusetts Institute of Technology Board. Written informed consent to participate in this study was provided by the participants’ legal guardian/next of kin.

## Author Contributions

VK made substantial contributions to the analysis or interpretation of data; drafted and revised the work critically for important intellectual content. FK made substantial contributions to the conception of the work; the analysis or interpretation of data; and revised the work critically for important intellectual content. CR-G made substantial contributions to the conception of the work; the acquisition and interpretation of data for the work; and revised it critically for important intellectual content. AM-G, BC-C, AG-G, and TCL made substantial contributions to the acquisition and analysis of data for the work; and revised it critically for important intellectual content. CD-M and RC-J made substantial contributions to the design of the work and revised it critically for important intellectual content. MG-R and TDL conceptualized and designed the work, supervised acquisition of data, contributed to data analysis and interpretation; drafted the work and revised it critically for important intellectual content. All authors contributed to the article and approved the submitted version.

## Funding

This work was possible thanks to the funding awarded to complete this project by the Mexican Foundation for Dermatology, the Mexican Government Ministry of Taxes Program E022 Health Research and Technological Development, Massachusetts Institute of Technology International Science and Technology Initiatives (MISTI) Global Seed Funds, and Deutsche Forschungsgemeinschaft (DFG) research fellowship (KE 2408/1-1 to FK).

## Conflict of Interest

The authors declare that the research was conducted in the absence of any commercial or financial relationships that could be construed as a potential conflict of interest.

## Publisher’s Note

All claims expressed in this article are solely those of the authors and do not necessarily represent those of their affiliated organizations, or those of the publisher, the editors and the reviewers. Any product that may be evaluated in this article, or claim that may be made by its manufacturer, is not guaranteed or endorsed by the publisher.
