# Supplementary material for: The Skin Microbiome of Patients With Atopic Dermatitis Normalizes Gradually During Treatment
Source: Front Cell Infect Microbiol. 2021 Sep 24;11:720674. doi: 10.3389/fcimb.2021.720674 (PMC8498027; doi:10.3389/fcimb.2021.720674)

**Supplementary Figures**

**Supplementary Figure 1. Flowchart of patient enrollment and clinical data analysis.**

**
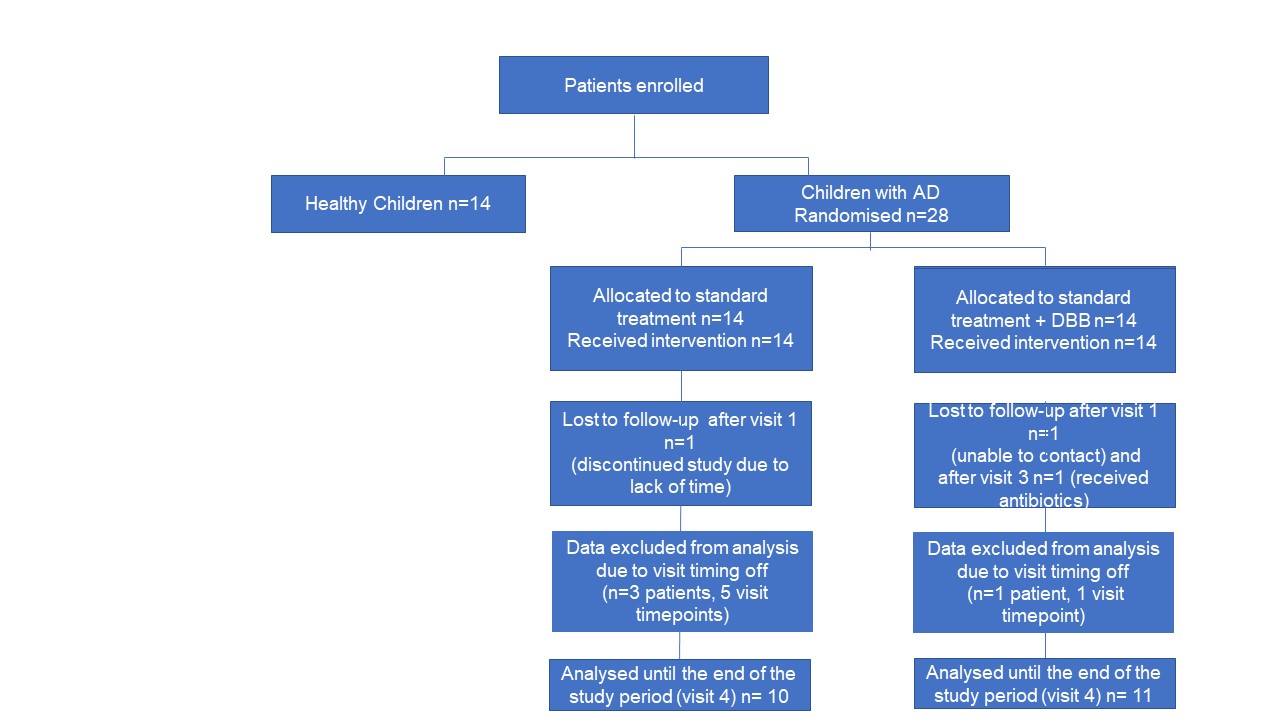
**

**Supplementary Figure 2. Differences in composition and diversity between treatment groups.** (**a**) Patients treated with DBB contain little to no taxa of the genus *Micrococcus*, whereas patients that did not receive treatment with DBB had little to no *S. capitis* in their microbiomes, in addition to differences in the relative abundance of S. aureus between the two groups.(**b**,**c**) Both treatment groups displayed a significant decrease in the relative abundance of *S. aureus* by visit 4 (3 months after initial visit). (**d**) There were no significant differences in alpha diversity (Shannon) between the two treatment groups, though the group that received DBB tended to have a slightly greater alpha diversity by later visits.

**
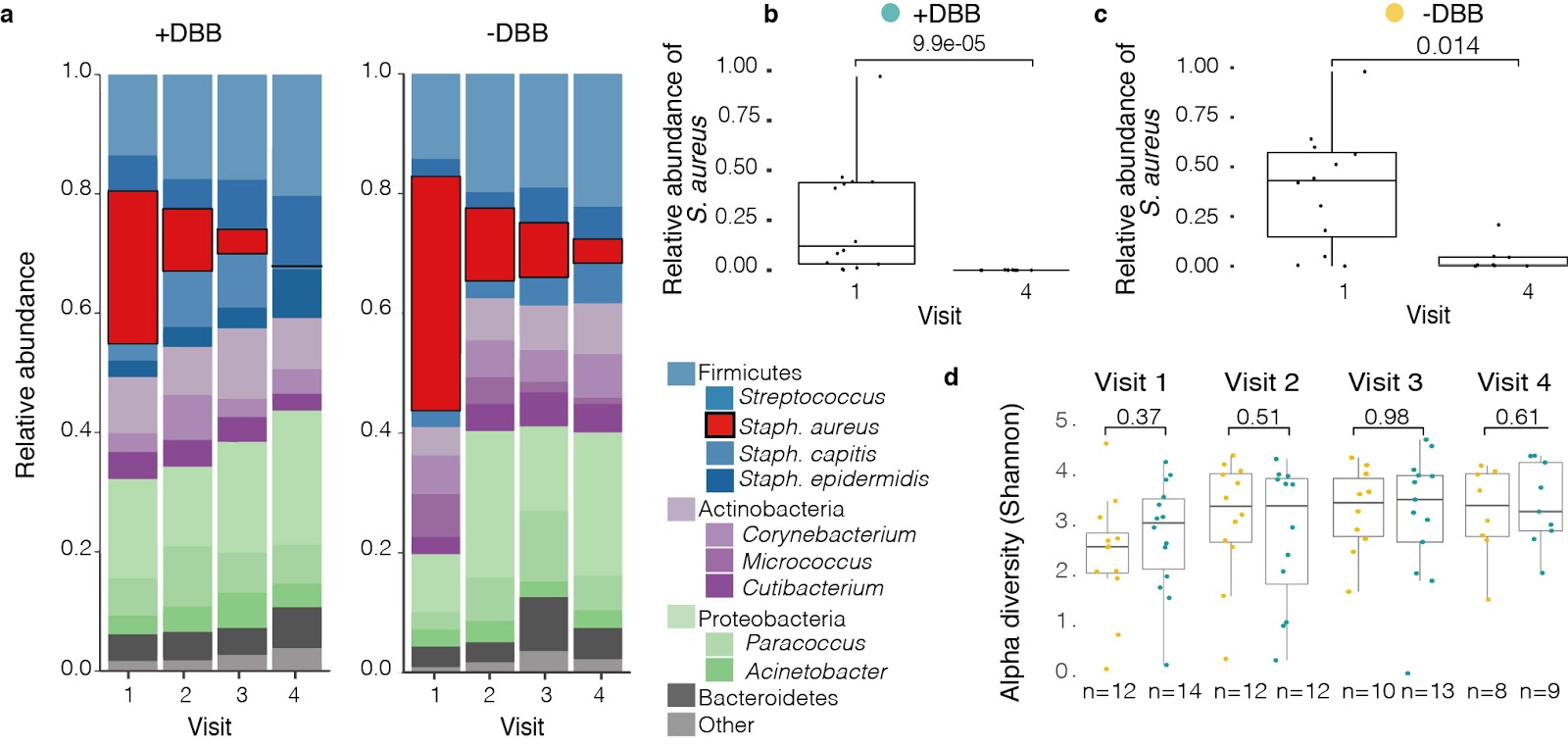
**

**Supplementary Figure 3. Health-like staphylococci are inversely correlated with disease severity.** (**a**) *S. epidermidis*, a bacterium thought to be associated with a “healthy” or “health-like” microbiota, is mildly correlated with SCORAD, largely driven by the presence of outliers, Spearman’s rho: -0.217, p=0.04 (**b**) *S. capitis* is not significantly correlated with SCORAD, Spearman’s rho: -0.033, p=0.758. (**c**) *S. hominis* is mildly correlated with SCORAD Spearman’s rho: -0.227, p=0.031. (**d**) The relative abundance of all staphylococcal species, excluding *S. aureus*, is not significantly correlated with SCORAD Spearman’s rho: 0.005, p=0.964


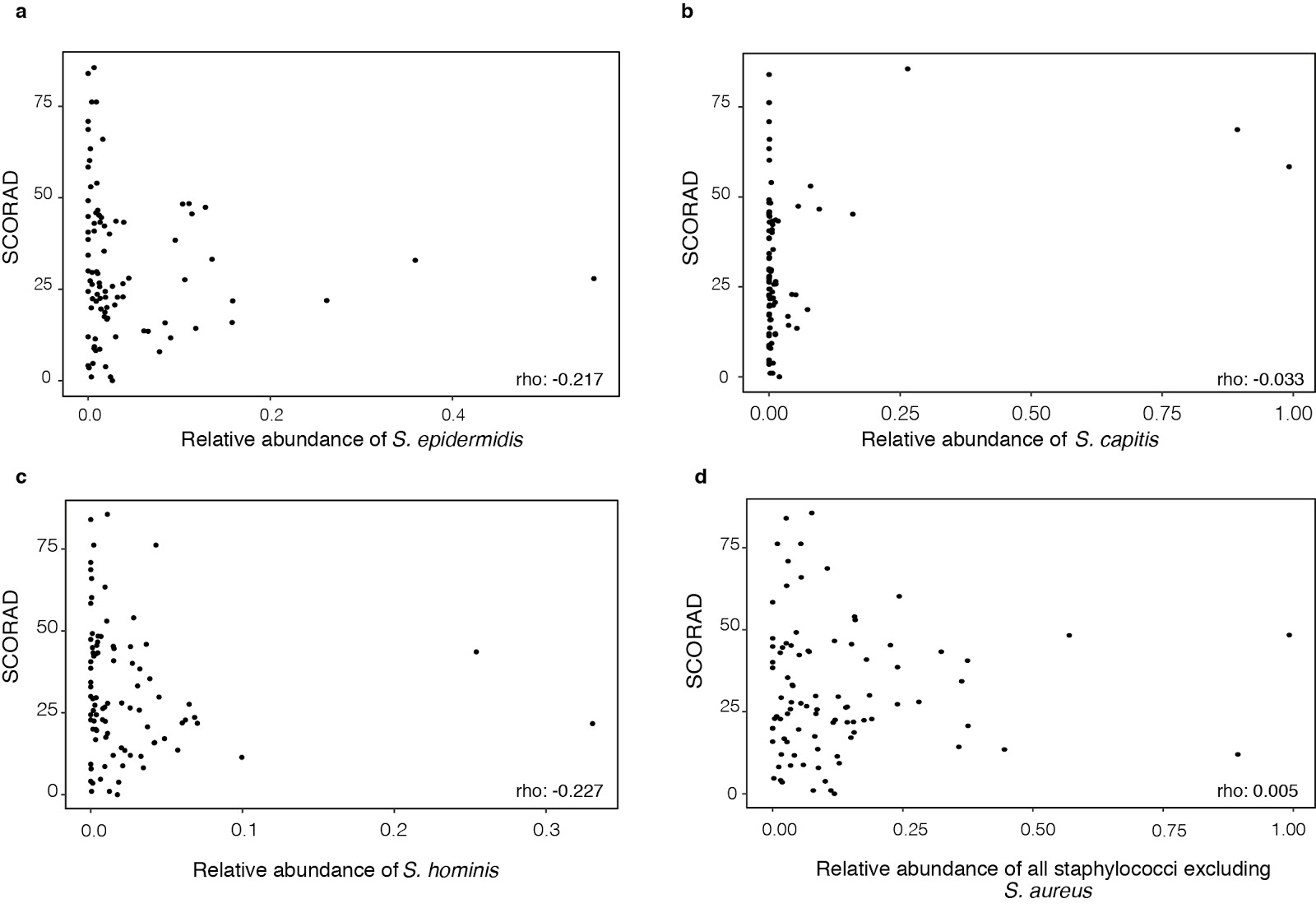


**Supplementary Figure 4. Community composition underlying *S. aureus* dominance is similarly diverse in patients and controls.** (**a**) Community composition of skin microbiome of healthy controls and AD patients with *S. aureus* removed. Taxa present at high abundance (> 5%) are similar between the two groups. Baseline samples with an average of 300 reads or less following *S. aureus* removal (n=2) were discounted and samples were merged into subject-level groupings. (**b**) Shannon diversity (alpha) is inversely correlated with disease severity (p<0.001) in AD patients when *S. aureus* dominates the community at likely lesional sites (**c**) Shannon diversity (alpha) remains correlated with disease severity in AD patients when *S. aureus* is removed from community structure, although much less so (p<0.001). (**d**) Distribution of alpha diversity between healthy controls and AD patients is not significantly different once *S. aureus* is removed.


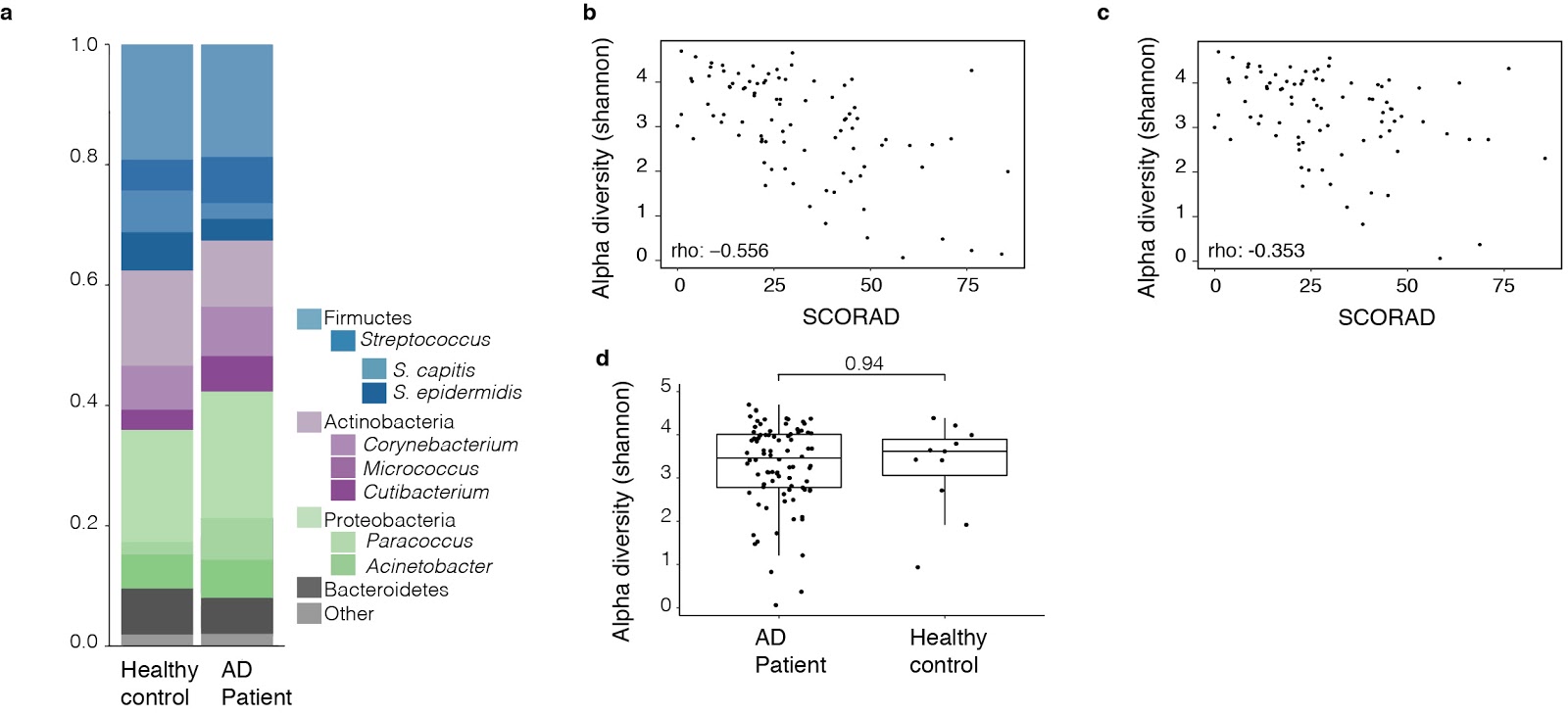

Supplement: Supplementary file 1 [file DataSheet_1.docx]
